# Core–Shell Ni/ZSM-5@SiO_2_ Bifunctional Catalysts for Enhanced *n*-Heptane Hydroisomerization via Regulated Metal–Acid Synergy

**DOI:** 10.3390/molecules31152704

**Published:** 2026-08-04

**Authors:** Xiuli Dong, Zhuoyan Li, Qinghua Du, Yingjun Wang, Yisi Zhou, Dianyou Yu, Ting Huang, Yanhua Suo

**Affiliations:** 1 College of Chemistry and Chemical Engineering, Northeast Petroleum University, Daqing 163318, China; dongxiuli@nepu.edu.cn (X.D.); 15710585567@163.com (Z.L.); huang_ting1998@126.com (T.H.); 2 China National Petroleum Corporation (CNPC) Daqing Oilfield Company, Daqing 163453, China; duqinghua6572@163.com; 3 College of Chemical Engineering and Technology, Yantai Nanshan University, Yantai 256713, China; wangying-jun@163.com; 4 Daqing Petrochemical Research Center, PetroChina Petrochemical Research Institute, Daqing 163714, China; zys459@petrochina.com.cn; 5 Department of Civil Engineering, Dalian University of Technology, Dalian 116024, China; yudianyou1992@163.com

**Keywords:** *n*-heptane hydroisomerization, ZSM-5, core-shell, SiO_2_, bifunctional catalysts

## Abstract

Improving gasoline octane number while reducing aromatics is crucial for clean fuel production. Herein, a core–shell 2% Ni/ZSM-5-138@SiO_2_-1 bifunctional catalyst was constructed by coating ZSM-5 with an inert SiO_2_ shell followed by Ni impregnation. The amorphous SiO_2_ shell effectively regulated the external surface acidity while preserving the MFI framework and hierarchical pore structure, leading to improved metal–acid synergy and suppressed undesired cracking. Under the optimized reaction conditions, the catalyst achieved an *n*-heptane conversion of 91.1% and an isomer selectivity of 56.7%. The enhanced catalytic performance is attributed to the synergistic optimization of surface acidity, mass transport, and metal–acid cooperation induced by the core–shell architecture. This work provides a practical strategy for designing efficient bifunctional catalysts for *n*-heptane hydroisomerization.

## 1. Introduction

The continuous growth in global industrialization and transportation has led to an increasing demand for petroleum-derived fuels, thereby intensifying the need for high-quality clean gasoline. Meanwhile, increasingly stringent environmental regulations have imposed severe restrictions on the contents of aromatics, olefins, and sulfur compounds in commercial gasoline, driving the development of advanced clean-fuel production technologies [1,2,3]. Among the various gasoline upgrading processes, the isomerization of linear alkanes has attracted considerable attention because it enables the conversion of low-octane *n*-paraffins into high-octane branched isomers without altering the carbon number of the feedstock. Consequently, this process represents one of the most effective approaches for improving gasoline octane ratings while reducing the dependence on aromatic blending components [4,5]. Isomerate gasoline is characterized by a high-octane number, low sulfur content, and negligible concentrations of aromatics and olefins, making it an indispensable blending component for the production of environmentally friendly gasoline with improved low-temperature performance [6]. With the gradual depletion of light petroleum resources, the feedstocks for isomerization have progressively shifted from C_5_–C_6_ *n*-paraffins to heavier C_7_^+^ hydrocarbons [7]. Among them, *n*-heptane, a representative C_7_ linear alkane with a research octane number (RON) close to zero, has become an ideal model compound for investigating hydroisomerization catalysts. Efficient conversion of *n*-heptane into highly branched isoheptanes can significantly improve gasoline octane quality while enhancing fuel utilization efficiency.

Hydroisomerization of *n*-heptane generally proceeds over bifunctional metal–acid catalysts via the well-established bifunctional reaction mechanism [8,9,10]. In bifunctional hydroisomerization catalysts, metallic sites generated after hydrogen activation promote the reversible dehydrogenation of *n*-heptane and subsequent hydrogenation of olefin intermediates, while Brønsted acid sites of zeolites facilitate carbocation formation and skeletal rearrangement. Therefore, bifunctional catalysts possessing efficient hydrogenation/dehydrogenation capability together with appropriate acidity are essential for achieving high hydroisomerization performance. Specifically, *n*-heptane is initially dehydrogenated on the metal sites to produce olefinic intermediates, which subsequently migrate to the zeolite BASs and undergo protonation, skeletal rearrangement, and isomerization to generate mono-branched, di-branched, and multi-branched olefins [10]. These intermediates then diffuse back to the metal sites for hydrogenation, ultimately yielding the corresponding branched alkanes [9]. The catalytic activity and product selectivity are therefore strongly governed by the balance between the metal and acid functions, as well as their synergistic regulation of the generation, diffusion, and transformation of reaction intermediates. Accordingly, rational catalyst design aimed at optimizing the metal–acid synergy while suppressing undesired cracking reactions remains one of the central challenges in *n*-heptane hydroisomerization.

Among various acidic zeolites, ZSM-5 has been extensively investigated as a promising support for hydroisomerization owing to its three-dimensional interconnected 10-membered-ring channel system, tunable acidity, and excellent thermal stability [11,12,13]. The shape-selective pore architecture effectively regulates the diffusion and transformation of reaction intermediates, thereby favoring the formation of mono-branched and di-branched isomers. In particular, high-silica ZSM-5 (e.g., Si/Al = 138) possesses a moderate Brønsted acid density and relatively mild acid strength, which can suppress excessive cracking to a certain extent and thus exhibits considerable potential for *n*-heptane hydroisomerization. Nevertheless, the external surface acid sites of ZSM-5 are not subject to shape-selective constraints, allowing reaction intermediates to undergo non-selective cracking, condensation, and coke formation on the external surface [14]. These side reactions not only reduce isomerization selectivity but also accelerate catalyst deactivation, thereby limiting the overall catalytic performance. To address this issue, constructing an inert shell to regulate the external surface acidity has emerged as an effective modification strategy [15,16]. An amorphous SiO_2_ shell can selectively passivate the external acid sites while preserving the intrinsic acidity within the zeolite channels, thereby suppressing undesired non-shape-selective reactions, optimizing the acid-site distribution, and improving the selectivity and yield of isomerization products. Moreover, the excellent chemical and thermal stability of SiO_2_ provides additional structural robustness for long-term catalytic operation.

Motivated by these considerations, a series of core–shell structured Ni/ZSM-5@SiO_2_ bifunctional catalysts were constructed via hydrothermal growth of an amorphous SiO_2_ shell on ZSM-5 followed by Ni impregnation. By systematically tuning the SiO_2_ shell thickness and the Si/Al ratio of the ZSM-5 core, the effects of the core–shell architecture on the pore structure, acid-site distribution, metal dispersion, and catalytic performance were comprehensively investigated. Particular attention was devoted to elucidating how the core–shell structure regulates the metal–acid synergy and consequently enhances the hydroisomerization selectivity of *n*-heptane. This work provides new insights into the rational design of high-performance bifunctional catalysts for *n*-heptane hydroisomerization.

## 2. Results and Discussion

### 2.1. XRD Characterization

**Figure 1:**
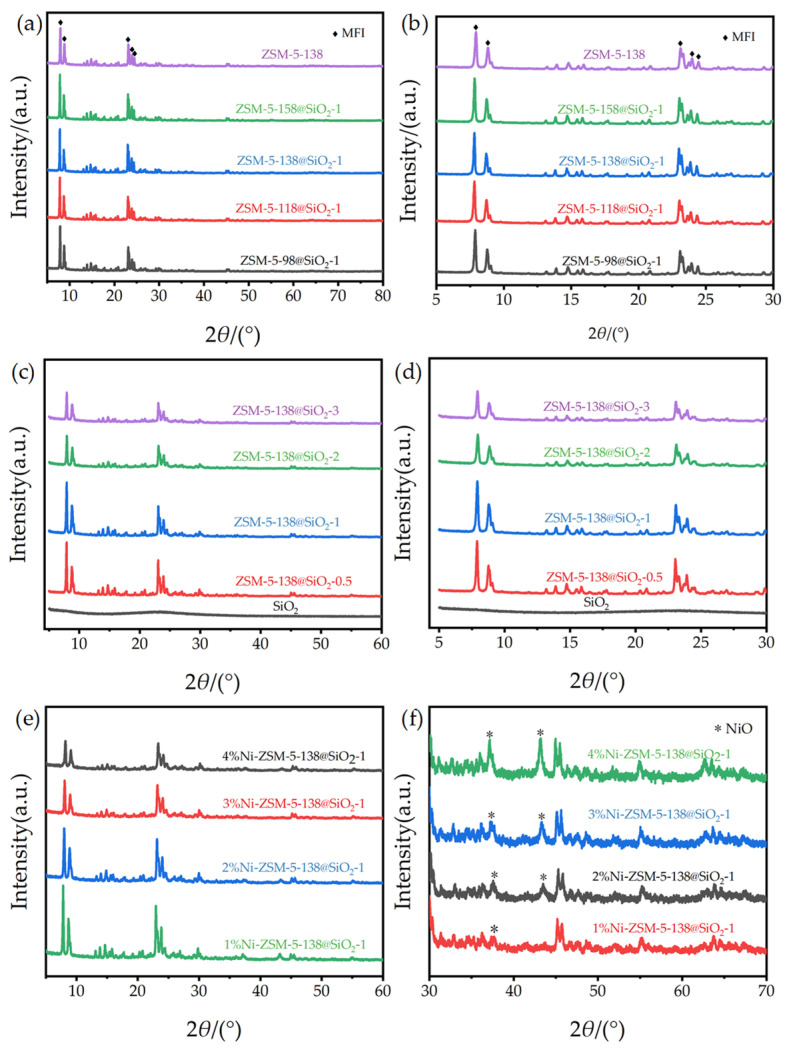
XRD patterns of different samples: (**a**) ZSM-5-x-SiO_2_-1; (**b**) enlarged XRD patterns of ZSM-5-x-SiO_2_-1; (**c**) ZSM-5-138-SiO_2_-y; (**d**) enlarged XRD patterns of ZSM-5-138-SiO_2_-y; (**e**) γ% Ni/ZSM-5-x@SiO_2_-y; (**f**) enlarged XRD patterns of γ% Ni/ZSM-5-x@SiO_2_-y.

Figure 1a,b present the wide-angle X-ray Diffraction (XRD) patterns of ZSM-5-138 and the ZSM-5@SiO_2_ core–shell zeolites with different Si/Al ratios. To better distinguish weak structural variations, an enlarged inset of the characteristic diffraction region was added. The diffraction peaks located at 2*θ* = 7.9°, 8.8°, and 22–25° can be indexed to the (101), (020), (501), (151), and (303) crystal planes, respectively, which are characteristic reflections of the MFI zeolite framework [17]. After SiO_2_ coating, all samples retained the characteristic diffraction peaks of the MFI structure without the appearance of any additional crystalline phases, indicating that the hydrothermal coating process did not alter the crystalline framework of ZSM-5 and that the structural integrity of the zeolite core was well preserved.

To further evaluate the influence of the SiO_2_ shell, the XRD patterns of the core–shell zeolites prepared with different TEOS/ZSM-5 mass ratios, together with that of pure SiO_2_, were compared (Figure 1c,d). In the absence of ZSM-5, the SiO_2_ sample exhibited only a broad diffraction band centered at approximately 2*θ* = 22°, confirming the amorphous nature of the silica formed during the hydrothermal process. Although the MFI framework remained intact after SiO_2_ coating, the diffraction intensity of the characteristic ZSM-5 peaks gradually decreased with increasing SiO_2_ content. This attenuation is mainly attributed to the progressive thickening of the amorphous SiO_2_ shell, which weakens the diffraction signal from the crystalline ZSM-5 core, rather than to any phase transformation or structural collapse of the zeolite framework [18]. The appearance of a broad amorphous silica-related feature suggests the introduction of an amorphous SiO_2_ phase, while maintaining the structural stability of the zeolite framework.

The XRD patterns of the catalysts with different Ni loadings are shown in Figure 1e,f. With increasing Ni loading, the characteristic diffraction peaks of ZSM-5 became slightly weaker, suggesting that the introduction of Ni exerted a minor influence on the crystallinity of the zeolite without disrupting the MFI framework. When the Ni loading reached 1 wt%, a weak diffraction peak assigned to NiO appeared at approximately 2*θ* = 43.4°, indicating the formation of detectable crystalline NiO species. As the Ni loading further increased to 2–4 wt%, the diffraction peaks located at approximately 37.2° and 43.4° became progressively more intense, implying that excessive Ni loading promoted the aggregation of Ni species and the formation of larger crystalline NiO particles.

### 2.2. FT-IR Characterization

**Figure 2:**
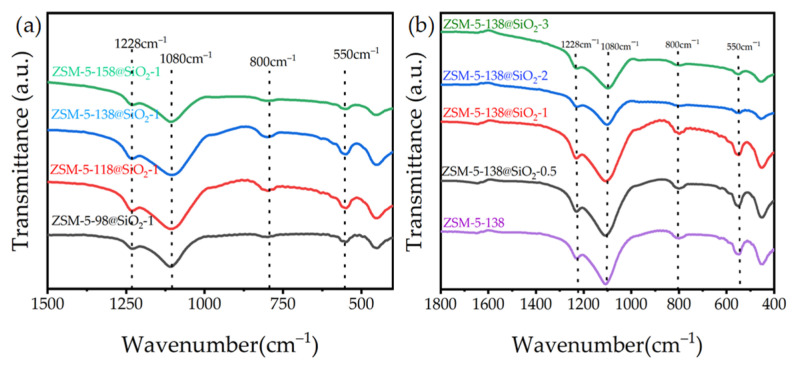
FT-IR spectra of different samples: (**a**) ZSM-5-x-SiO_2_-1; (**b**) ZSM-5-138-SiO_2_-y.

Figure 2 shows the Fourier Transform Infrared (FT-IR) spectra of the prepared samples. The absorption bands located at approximately 550 and 800 cm^−1^ are assigned to the characteristic vibration of the five-membered rings in the MFI framework and the symmetric stretching vibration of Si–O–Si bonds, respectively. The bands centered at 1080 and 1228 cm^−1^ correspond to the asymmetric stretching vibrations of Si–O–Si and Si–O–Al linkages, respectively, confirming that all samples retained the characteristic framework structure of ZSM-5.

As shown in Figure 2a, the intensity of the Si–O–Si-related bands increased slightly with increasing Si/Al ratio, whereas the contribution of the Si–O–Al vibration gradually decreased. This behavior is attributed to the reduced framework Al content and the corresponding increase in the proportion of Si–O–Si linkages within the zeolite framework.

The influence of the SiO_2_ shell was further investigated by varying the TEOS dosage, as presented in Figure 2b. With increasing TEOS content, the characteristic absorption bands associated with the ZSM-5 framework gradually weakened. This attenuation is mainly attributed to the progressive growth of the amorphous SiO_2_ shell on the external surface of ZSM-5, which partially shields the infrared vibration signals originating from the zeolite framework rather than indicating any destruction of the MFI structure. This interpretation is further supported by the XRD results, where the characteristic diffraction peaks of ZSM-5 remained unchanged after SiO_2_ coating. The above observations suggest that SiO_2_ is likely deposited on the external surface of ZSM-5 crystals.

### 2.3. Textural Properties

**Figure 3:**
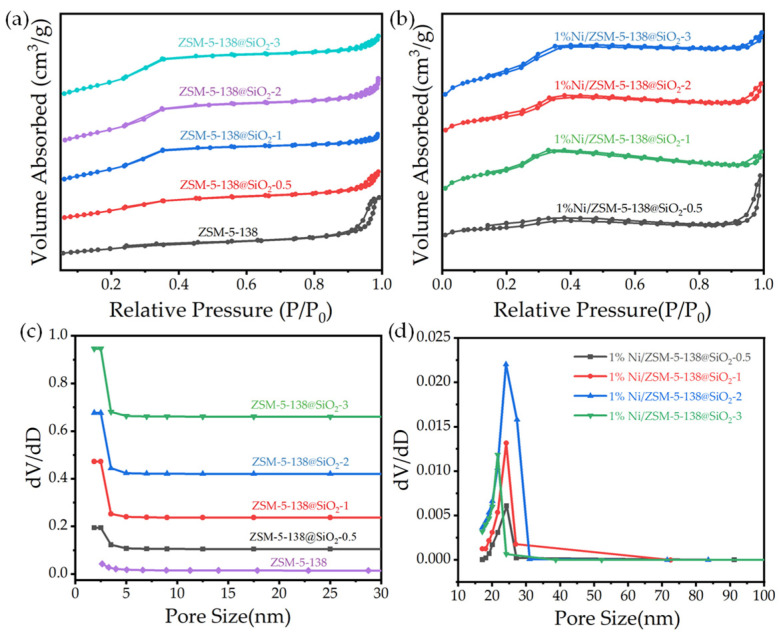
Nitrogen adsorption–desorption isotherms and pore size distributions of different samples: (**a**) ZSM-5-138-SiO_2_-y; (**b**) 1% Ni/ZSM-5-138-SiO_2_-y; (**c**) pore size distributions of ZSM-5-138-SiO_2_-y; (**d**) pore size distributions of 1% Ni/ZSM-5-138-SiO_2_-y.

Figure 3 presents the N_2_ adsorption–desorption isotherms and pore size distribution curves of ZSM-5, ZSM-5@SiO_2_, and Ni/ZSM-5@SiO_2_ catalysts. Pristine ZSM-5 exhibits a typical type IV adsorption–desorption isotherm, indicating the presence of intercrystallite mesopores in addition to its intrinsic microporous structure [19]. After SiO_2_ coating and subsequent Ni loading, all samples display a pronounced H1-type hysteresis loop at relative pressures (P/P_0_) above 0.4, suggesting the formation of relatively uniform mesoporous structures [20].

As shown in Figure 3c,d, the pore size distribution is mainly centered within the 20–30 nm range, while the intrinsic micropores of the ZSM-5 framework are well preserved. These results demonstrate that the as-prepared core–shell catalysts possess a hierarchical micro–mesoporous architecture, which is expected to facilitate the diffusion and transport of reactant and product molecules during the hydroisomerization reaction.

With increasing TEOS content, the nitrogen adsorption capacity gradually increased, indicating that the introduction of the SiO_2_ shell effectively enhanced the mesopore surface area and pore volume (Table 1). However, when the TEOS/ZSM-5 mass ratio was further increased to 3, the total specific surface area decreased from 500.16 to 479.81 m^2^·g^−1^. Meanwhile, the mesopore surface area initially increased from 394.07 to 453.12 m^2^·g^−1^ and subsequently declined to 442.29 m^2^·g^−1^, whereas the micropore surface area decreased markedly from 106.09 to 37.53 m^2^·g^−1^. Although only a small amount of silica was introduced, the microporous surface area decreased significantly. This behavior is possibly related to the extremely narrow aperture of ZSM-5 channels. Silica species deposited at the external surface can partially block pore mouths, resulting in reduced accessibility of internal micropores during adsorption measurements. Such pore-mouth modification is expected for silica coating and does not necessarily indicate collapse of the zeolite framework. These results indicate that an appropriate SiO_2_ coating promotes the development of mesoporous channels and improves mass transport, whereas an excessively thick SiO_2_ shell may partially block pore entrances or reduce pore connectivity, thereby decreasing the accessibility of the intrinsic microporous network and hindering the diffusion of reactant molecules toward the active sites.

Compared with the corresponding supports, the Ni-loaded catalysts exhibit lower specific surface areas, total pore volumes, and mesopore volumes, whereas the adsorption–desorption isotherm profiles and H1-type hysteresis loops remain essentially unchanged. No direct evidence suggests that Ni species generate mesopores through framework destruction. The different mesoporous characteristics of Ni-containing samples may originate from the modified surface chemistry during silica deposition, which influences the development or accessibility of external pore structures. In addition, both the total pore volume and the average pore diameter decrease after Ni loading. Combined with the TEM and XRD results, these observations suggest that partial aggregation of Ni species occurs within the mesoporous region, leading to a reduction in the textural parameters. Therefore, the deposited Ni species are considered to be predominantly distributed within the SiO_2_ shell and its associated mesoporous network.

### 2.4. SEM Characterization

**Figure 4:**
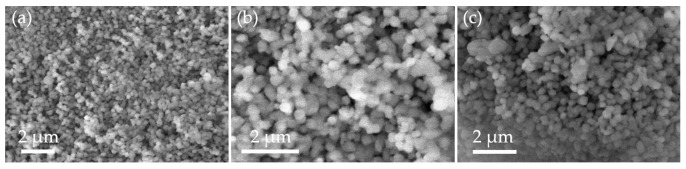
SEM images of different samples: (**a**) ZSM-5-138; (**b**) ZSM-5-138@SiO_2_-1; (**c**) 2% Ni/ZSM-5-138@SiO_2_-1.

Figure 4 presents the Scanning Electron Microscopy (SEM) images of ZSM-5-138, ZSM-5-138@SiO_2_-1, and 2% Ni/ZSM-5-138@SiO_2_-1. As shown in Figure 4a, the pristine ZSM-5-138 sample consists of relatively uniform spherical particles with a certain degree of interparticle aggregation while maintaining an intact crystal morphology. After SiO_2_ coating (Figure 4b), the spherical morphology of the zeolite particles was well preserved, and no obvious crystal fracture or structural collapse was observed, indicating that the hydrothermal coating process had a negligible influence on the morphology of the ZSM-5 crystals. Meanwhile, slight interparticle bridging and aggregation became apparent, which can be attributed to the continuous growth of the amorphous SiO_2_ layer on the external surface of the zeolite particles. This observation provides preliminary evidence for the successful formation of a core–shell architecture. As shown in Figure 4c, the overall morphology of the catalyst remained essentially unchanged after loading 2 wt% Ni, exhibiting a morphology similar to that of ZSM-5@SiO_2_-1. This indicates that all samples maintain the typical morphology of ZSM-5 crystals after silica modification and Ni incorporation. No obvious particle collapse or severe aggregation is observed, indicating that the modification process does not destroy the original zeolite morphology.

### 2.5. TEM and Elemental Mapping Analyses

**Figure 5:**
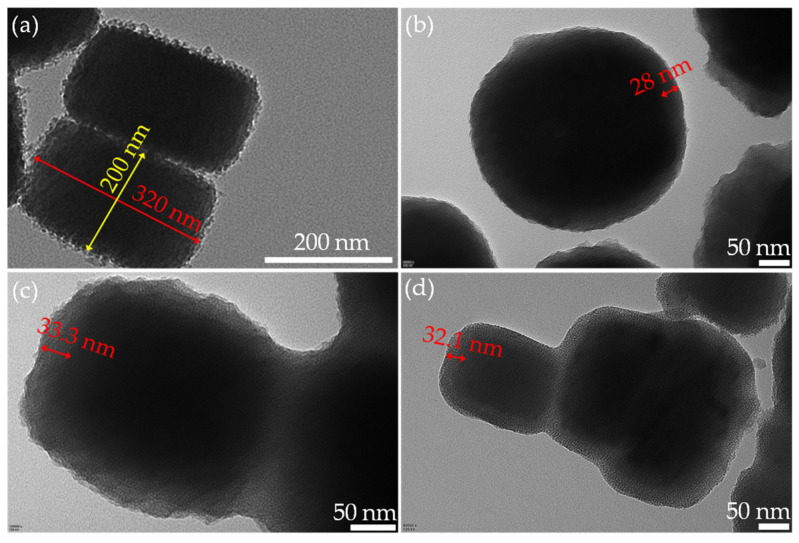
TEM images and elemental mapping of different samples: (**a**) ZSM-5-138; (**b**) ZSM-5-138@SiO_2_-1; (**c**) ZSM-5-138@SiO_2_-2; (**d**) ZSM-5-138@SiO_2_-3.

Figure 5 presents the electron microscopy images of ZSM-5-138 and ZSM-5-138@SiO_2_ samples. As shown in Figure 5a, the parent ZSM-5 crystals exhibit a typical hexagonal prism morphology with a relatively uniform particle size, and the dimensions of the hexagonal prisms are approximately 320 × 200 nm. Figure 5b–d display the TEM images of ZSM-5@SiO_2_ samples synthesized with different m_TEOS_: m_ZSM-5_ ratios (1, 2, and 3). The ZSM-5@SiO_2_ particles clearly exhibit core–shell structures with spherical or prism-like morphologies, indicating that the SiO_2_ layer is uniformly coated on the external surface of ZSM-5 crystals. With increasing TEOS dosage, the SiO_2_ shells gradually become thicker and tend to interconnect due to the enhanced silica deposition. The shell thickness of the core–shell catalysts increases progressively with increasing m_TEOS_: m_ZSM-5_ ratio, from 28 nm to 33.1 nm. However, when the m_TEOS_: m_ZSM-5_ ratio further increases to 3, the shell thickness remains nearly unchanged (32.1 nm), suggesting that excessive TEOS addition does not promote further SiO_2_ shell growth, possibly due to the limited availability of accessible surface sites for silica deposition.

**Figure 6:**
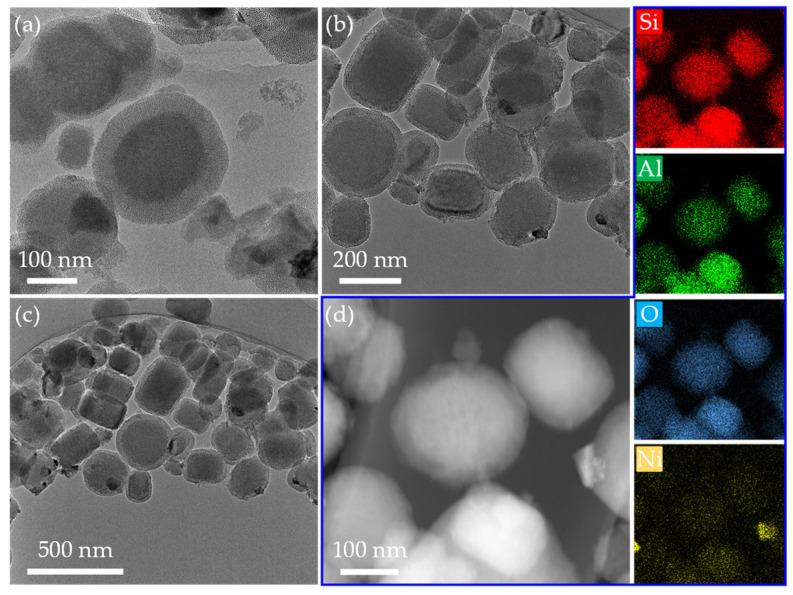
TEM images (**a**–**c**) and elemental mapping (**d**) of 2% Ni/ZSM-5-138@SiO_2_-1.

Figure 6 presents the TEM images and corresponding EDS elemental mapping results of the 2% Ni/ZSM-5-138@SiO_2_-1 catalyst. As shown in Figure 6a–c, the catalyst retains a well-defined spherical morphology, which is consistent with the SEM observations, indicating that neither the hydrothermal SiO_2_ coating process nor the subsequent Ni impregnation altered the overall morphology of the catalyst. In addition, a small number of high-contrast nanoparticles can be observed on the external surface of several particles. Combined with the XRD results, these nanoparticles are reasonably assigned to locally crystallized NiO species, suggesting that a limited fraction of Ni species underwent aggregation during catalyst preparation.

The elemental mapping results are shown in Figure 6d. Silicon, aluminum, oxygen, and nickel are uniformly distributed throughout the catalyst with distinct spatial distributions. Aluminum is predominantly concentrated in the interior region of the particles, whereas silicon is distributed over the entire catalyst, confirming that the ZSM-5 core remained structurally intact after SiO_2_ coating. In contrast, nickel is mainly enriched in the peripheral region corresponding to the SiO_2_ shell, with only a few localized regions exhibiting relatively higher Ni intensity. This distribution indicates that the deposited Ni species are preferentially located within the SiO_2_ shell while maintaining a relatively high dispersion. The spatial distributions of Si, Al, and Ni collectively provide strong evidence for the successful construction of the core–shell architecture.

### 2.6. NH_3_-TPD

**Figure 7:**
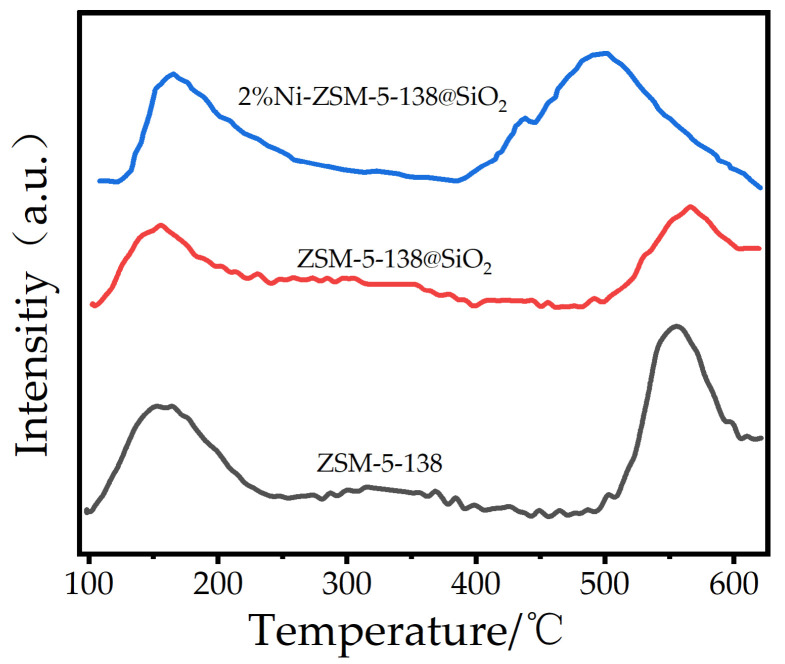
NH_3_-TPD profiles of different samples.

The acidity of ZSM-5-138, ZSM-5-138@SiO_2_-1, and 2% Ni/ZSM-5-138@SiO_2_-1 was investigated by NH_3_ Temperature-Programmed Desorption (NH_3_-TPD), and the corresponding acidity parameters are summarized in Table 2. As shown in Figure 7, all samples exhibit two well-defined NH_3_ desorption peaks. The low-temperature peak at approximately 160–180 °C is assigned to weak acid sites, whereas the high-temperature peak located at 500–560 °C corresponds to strong acid sites, in agreement with previous reports [21]. The latter is generally attributed to NH_3_ ad-sorbed on Brønsted acid sites (Si–OH–Al), reflecting the characteristics of the strong Brønsted acidity in the zeolite framework.

Compared with the parent ZSM-5, both the low- and high-temperature desorption peak areas of ZSM-5@SiO_2_-1 decrease markedly, and the total acidity decreases from 0.25 to 0.14 mmol/g, whereas the positions of the desorption peaks remain essentially unchanged. These results indicate that the introduction of the SiO_2_ shell effectively reduces the number of accessible acid sites without significantly altering their intrinsic acid strength. Combined with the FT-IR and XRD analyses, it can be inferred that the amorphous SiO_2_ shell predominantly passivates the acid sites exposed on the external surface of ZSM-5 while preserving the integrity of the MFI framework and its intrinsic Brønsted acidity. Such selective regulation of the external surface acidity is expected to suppress non-shape-selective cracking reactions and thereby improve the selectivity toward hydroisomerization.

After loading 2 wt% Ni, both the weak- and strong-acid desorption peak areas in-crease, resulting in a total acidity of 0.38 mmol/g. It should be noted that the NH_3_ uptake represents the overall acidity of the catalyst, including Brønsted acid sites, Lewis acid sites, and additional adsorption sites associated with supported Ni species. The enhanced acidity after Ni loading can be associated with the formation of Ni-related Lewis acidic centers. The positively charged Ni species interacting with oxygen atoms of the zeolite framework can provide additional electron-deficient sites for NH_3_ adsorption. Meanwhile, surface hydroxyl groups generated during Ni precursor decomposition may also contribute to ammonia adsorption. These effects collectively increase the measured total acidity in NH_3_-TPD analysis. Meanwhile, the high-temperature desorption peak shifts slightly toward lower temperatures, indicating a moderate decrease in the average strength of the strong acid sites. This result suggests that the introduction of Ni modifies the surface acid environment of the catalyst, leading to an increase in the number of accessible acid sites while slightly weakening their average acid strength. Such changes are expected to optimize the balance between the metallic and acidic functions, thereby facilitating the hydroisomerization of *n*-heptane while suppressing undesired cracking reactions.

### 2.7. XPS Analysis

**Figure 8:**
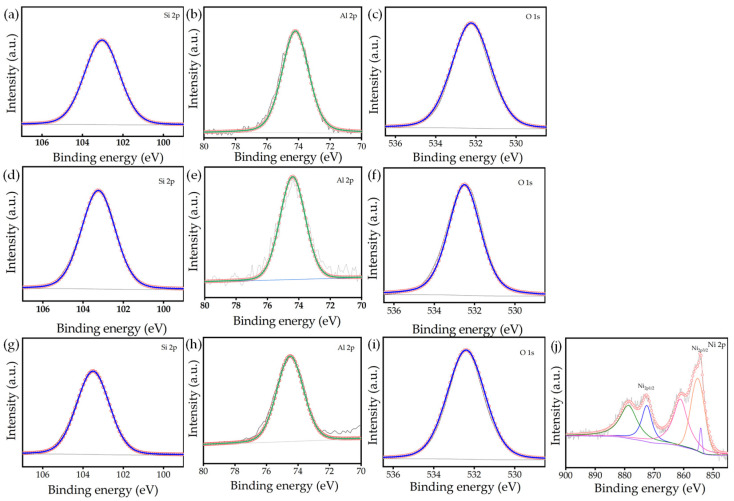
XPS spectra of (**a**–**c**) ZSM-5-138 (Si 2p, Al 2p, O 1s), (**d**–**f**) ZSM-5-138@SiO_2_-1 (Si 2p, Al 2p, O 1s), and (**g**–**j**) 2%Ni/ZSM-5-138@SiO_2_-1 (Si 2p, Al 2p, O 1s, Ni 2p) (Reaction temperature: 300 °C; Reaction time: 30 min).

To further investigate the surface elemental composition and chemical states of the catalysts, X-ray Photoelectron Spectroscopy (XPS) was performed on ZSM-5-138, ZSM-5-138@SiO_2_-1, and 2% Ni/ZSM-5-138@SiO_2_-1, and the corresponding spectra are shown in Figure 8. The high-resolution Ni 2p spectrum of the 2% Ni/ZSM-5-138@SiO_2_-1 catalyst is presented in Figure 8j. The dominant Ni 2p_3/2_ peak located at approximately 855.2 eV is characteristic of Ni^2+^ species, which are generally assigned to NiO [22]. The accompanying satellite peaks centered at approximately 861.1 and 872.5 eV are typical shake-up satellite features of Ni^2+^ species arising from charge-transfer processes during photoemission [23], further confirming that Ni predominantly exists in the oxidized state. In addition, there is a Ni 2p^3/2^ peak belonging to NiO at 854.2 eV [24,25]. These results indicate that the deposited Ni is mainly present as oxidized Ni species and that its chemical environment is influenced by the interaction with the ZSM-5-138@SiO_2_-1 support. The XPS spectra were collected for the calcined catalysts; therefore, Ni species were mainly detected in the oxidized Ni^2+^ state. These results provide information regarding the surface distribution and metal–support interaction of Ni species before reduction. During catalytic evaluation, the H_2_ pretreatment converts NiO species into metallic Ni^0^ sites, which serve as the active centers for *n*-heptane dehydrogenation and olefin hydrogenation.

The O 1s spectra of the three catalysts are shown in Figure 8c,f,i. The dominant peak located at approximately 532 eV is assigned to lattice oxygen associated with Si–O–Si linkages in the zeolite framework [26,27], whereas the higher-binding-energy component is attributed to surface hydroxyl groups and chemisorbed oxygen species [28,29]. Slight shifts in the O 1s binding energy after SiO_2_ coating and Ni loading suggest that the local electronic environment of surface oxygen species is modified, indicating electronic interactions between the deposited Ni species and the support.

The Al 2p spectra (Figure 8b,e,h) exhibit characteristic peaks centered at approximately 74 eV, corresponding to framework Al–O–Si species, indicating that neither the hydrothermal coating process nor the subsequent Ni loading altered the coordination environment of framework aluminum [28]. Likewise, the Si 2p spectra (Figure 8a,d,g) display characteristic Si^4+^ peaks at approximately 103 eV, which are assigned to Si–O–Si bonds [30], confirming that the fundamental framework structure of ZSM-5-138 remains intact after catalyst modification. Compared with the Ni-free sample, the binding energies of the Si 2p, Al 2p, and O 1s signals exhibit slight positive shifts after Ni loading. These shifts indicate a redistribution of surface electron density caused by the interaction between Ni species and the ZSM-5-138@SiO_2_-1 support, suggesting the presence of electronic metal–support interactions that may influence the catalytic behavior during *n*-heptane hydroisomerization.

To further investigate the surface chemical composition and the effect of silica modification, XPS quantitative analysis was performed, and the corresponding results are summarized in Table 2. The XPS results show that the surface Al concentration decreased after silica modification, accompanied by an increase in the surface Si proportion. Since XPS is a surface-sensitive technique with a probing depth of only several nanometers, the reduced Al signal indicates that the external zeolite surface is partially covered by silica species, providing additional evidence for the formation of a silica-coated ZSM-5 structure. After Ni incorporation, a Ni signal of 0.95 at% was detected, confirming the successful introduction of Ni species onto the silica-modified zeolite surface.

### 2.8. Catalytic Performance

#### 2.8.1. Effect of SiO_2_ Shell Thickness on *n*-Heptane Hydroisomerization

**Figure 9:**
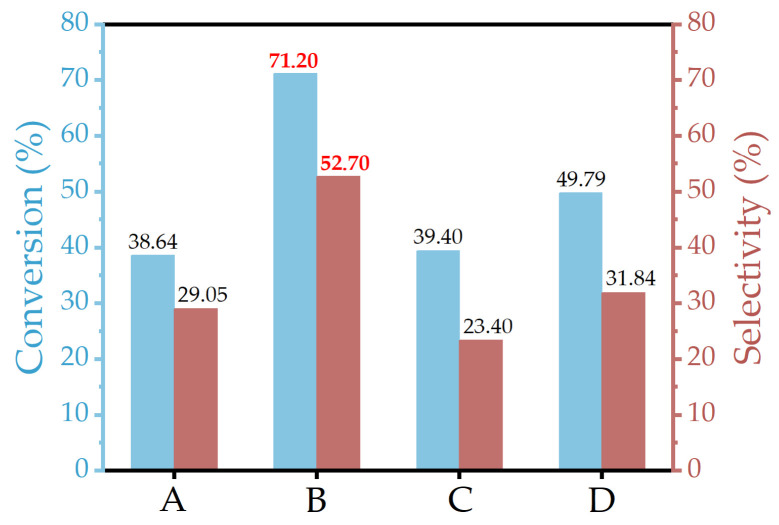
Catalytic performance as a function of the m_TEOS_/m_ZSM-5_ ratio: (A) 1%Ni/ZSM-5-118@SiO_2_-0.5, (B) 1%Ni/ZSM-5-118@SiO_2_-1, (C) 1%Ni/ZSM-5-118@SiO_2_, (D) 1%Ni/ZSM-5-118 (Reaction temperature: 300 °C; Reaction time: 30 min).

Figure 9 illustrates the catalytic performance of the catalysts with different SiO_2_ shell thicknesses for the hydroisomerization of *n*-heptane under identical reaction conditions. As the TEOS/ZSM-5 mass ratio increased, both the *n*-heptane conversion and isomer selectivity exhibited a typical volcano-shaped dependence on the SiO_2_ shell thickness. The catalyst prepared with a TEOS/ZSM-5 mass ratio of 1 exhibited the optimum performance, affording an *n*-heptane conversion of 71.2% and an isomer selectivity of 52.7%, which are 21.4% and 20.9% higher, respectively, than those obtained over the uncoated ZSM-5 catalyst. These results demonstrate that an appropriately designed SiO_2_ shell can substantially enhance the hydroisomerization performance, whereas either an insufficient or an excessively thick shell is detrimental to catalytic activity.

The structural characterization results provide insight into the origin of this behavior. According to the N_2_ adsorption–desorption analysis, the introduction of the SiO_2_ shell decreases the microporous surface area of ZSM-5 while simultaneously generating abundant mesoporous channels. Although partial coverage of the zeolite surface inevitably reduces the accessibility of some micropores, the newly formed mesoporous network significantly improves the diffusion of reactant molecules and reaction intermediates. More importantly, the enhanced mass transport facilitates the rapid desorption of isomerized products from the acid sites, thereby suppressing secondary cracking reactions. However, when the SiO_2_ shell becomes excessively thick, pore connectivity is partially reduced and diffusion resistance increases, resulting in a decline in catalytic activity.

Furthermore, the XRD and NH_3_-TPD results reveal that the SiO_2_ shell does not alter the MFI framework of ZSM-5 but instead selectively passivates the external acid sites, thereby optimizing the spatial distribution of acidity. By preserving the intrinsic Brønsted acid sites within the zeolite channels while suppressing non-shape-selective acid-catalyzed reactions occurring on the external surface, the core–shell architecture promotes the hydroisomerization pathway over undesired side reactions. Such selective regulation of the external surface acidity is also expected to mitigate coke formation associated with external acid sites, thereby contributing to the improved catalytic performance.

#### 2.8.2. Effect of the Si/Al Ratio of the ZSM-5 Core on *n*-Heptane Hydroisomerization

The acidity of the zeolite support is one of the key factors governing the hydroisomerization of *n*-heptane. Brønsted acid sites are responsible for the protonation of olefin intermediates and the subsequent skeletal rearrangement of carbenium ions, whereas excessive acid density or overly strong acid sites tend to promote undesirable cracking reactions [31]. Therefore, optimizing both the density and strength of the acid sites is essential for balancing the competing hydroisomerization and cracking pathways, thereby maximizing isomer yield [32].

**Figure 10:**
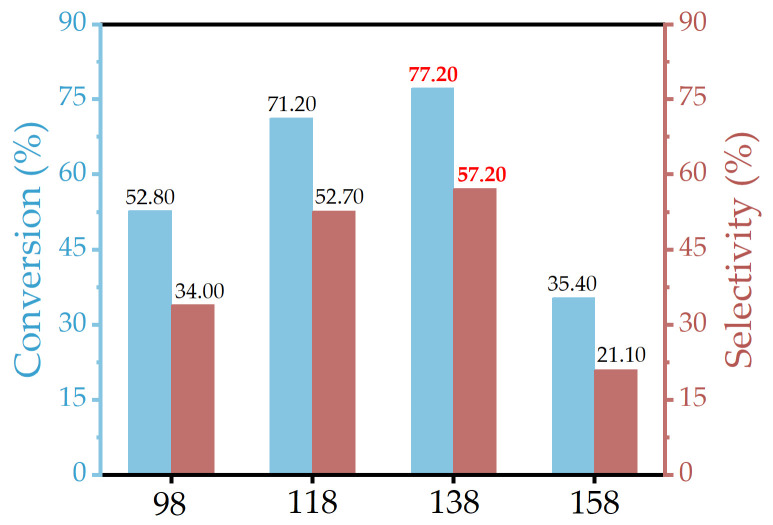
Catalytic performance as a function of the Si/Al molar ratio of the ZSM-5 core.

Figure 10 presents the catalytic performance of the catalysts with different Si/Al ratios under identical reaction conditions. As the Si/Al ratio increased, both the *n*-heptane conversion and isomer selectivity exhibited a typical volcano-shaped dependence on the framework composition. The catalyst with a Si/Al ratio of 138 delivered the highest catalytic performance, achieving an *n*-heptane conversion of 77.2% together with an isomer selectivity of 57.2%.

This behavior can be attributed to the gradual decrease in acid density with increasing Si/Al ratio. A moderate reduction in the number of strong Brønsted acid sites effectively suppresses excessive cracking while maintaining sufficient acidity to facilitate the protonation and skeletal rearrangement of olefin intermediates, thereby improving isomer selectivity. However, a further increase in the Si/Al ratio substantially decreases the concentration of Brønsted acid sites, limiting the formation and rearrangement of carbenium ion intermediates and consequently reducing the hydroisomerization rate. Combined with the structural characterization results, these findings indicate that the catalyst with a Si/Al ratio of 138 possesses an optimized balance between framework acidity and pore architecture. The preserved MFI framework together with the hierarchical pore structure provides favorable conditions for molecular diffusion, carbenium ion rearrangement, and rapid desorption of the isomerized products, ultimately leading to the highest hydroisomerization performance.

#### 2.8.3. Effect of Ni Loading on *n*-Heptane Hydroisomerization

**Figure 11:**
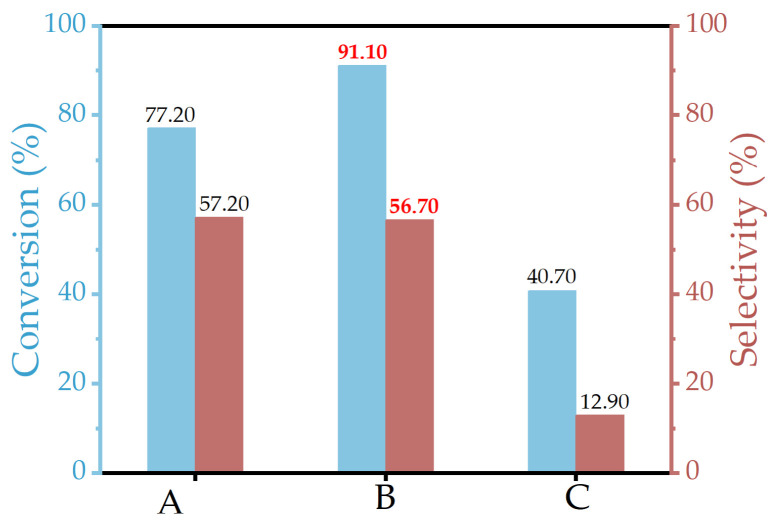
Catalytic performance as a function of Ni loading: (A) 1%Ni/ZSM-5-138@SiO_2_-1, (B) 2%Ni/ZSM-5-138@SiO_2_-1, (C) 3%Ni/ZSM-5-138@SiO_2_-1.

The metallic function plays a crucial role in bifunctional catalysts by catalyzing the dehydrogenation of *n*-heptane and the subsequent hydrogenation of isomerized olefin intermediates, thereby strongly influencing the overall hydroisomerization performance. Figure 11 shows the catalytic performance of catalysts with different Ni loadings under identical reaction conditions. As the Ni loading increased, the *n*-heptane conversion exhibited a typical volcano-shaped dependence on the metal content.

At Ni loadings below 2 wt%, the limited number of accessible metallic active sites restricts the dehydrogenation capability of the catalyst, resulting in insufficient generation of olefin intermediates and consequently lower hydroisomerization activity. Increasing the Ni loading to 2 wt% markedly enhances the catalytic performance, affording an *n*-heptane conversion of 91.1% and an isomer selectivity of 56.7%. These results indicate that an appropriate amount of Ni provides sufficient metallic active sites to efficiently promote both the dehydrogenation of *n*-heptane and the hydrogenation of isomerized olefin intermediates, thereby achieving a more favorable balance between the metallic and acidic functions and enhancing the overall hydroisomerization performance.

When the Ni loading exceeded 2 wt%, the catalytic activity gradually decreased. According to the XRD and TEM results, higher Ni loadings led to the appearance of crystalline NiO phases and partial aggregation of Ni species. Such aggregation decreases the dispersion of metallic active sites and reduces the number of accessible Ni sites participating in the hydrogenation–dehydrogenation cycle. Consequently, the balance between the metallic and acidic functions is disrupted, resulting in a deterioration of the hydroisomerization performance.

#### 2.8.4. Effect of Reaction Temperature on *n*-Heptane Hydroisomerization

**Figure 12:**
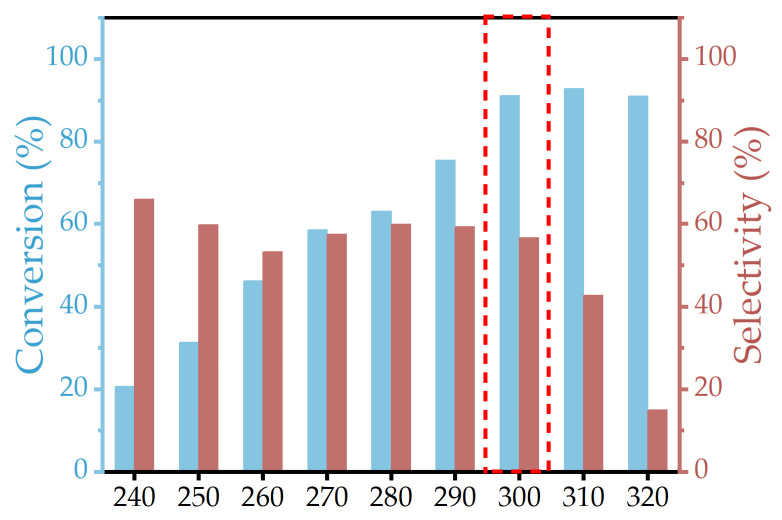
Catalytic performance of the 2%Ni/ZSM-5-138@SiO_2_-1 catalyst as a function of reaction temperature.

Figure 12 illustrates the effect of reaction temperature on the hydroisomerization performance of the 2% Ni/ZSM-5-138@SiO_2_-1 catalyst. As the reaction temperature increased from 240 to 320 °C, the *n*-heptane conversion continuously increased, whereas the isomer selectivity exhibited a volcano-shaped trend, initially increasing and then decreasing. At relatively low temperatures, the activation of *n*-heptane molecules and the dehydrogenation reaction occurring on the Ni active sites are kinetically limited, resulting in a low concentration of olefin intermediates available for subsequent protonation and skeletal rearrangement over the Brønsted acid sites. Consequently, the overall hydroisomerization activity remains relatively low. As the reaction temperature increases, both the dehydrogenation of *n*-heptane and the skeletal isomerization of carbenium ion intermediates are accelerated, thereby facilitating the bifunctional hydroisomerization cycle and leading to a continuous increase in *n*-heptane conversion.

The optimum catalytic performance was achieved at 300 °C, affording an *n*-heptane conversion of 91.1%, an isomer selectivity of 56.7%, and an isomer yield of 51.6%. This result indicates that an appropriate reaction temperature provides a favorable balance between the metallic and acidic functions while maintaining efficient molecular diffusion and intermediate conversion. Further increasing the reaction temperature resulted in a decline in isomer selectivity. This behavior can be attributed to the thermodynamic and kinetic characteristics of the competing reaction pathways. Although hydroisomerization is an exothermic reaction, cracking reactions become increasingly favorable at elevated temperatures because of their higher activation energies. Consequently, secondary cracking of the isomerized intermediates is promoted, leading to the formation of lighter hydro-carbons at the expense of the desired isomerized products. As a result, the selectivity toward isomerized products decreases despite the continued increase in *n*-heptane conversion.

#### 2.8.5. Catalytic Stability of 2% Ni/ZSM-5-138@SiO_2_-1

**Figure 13:**
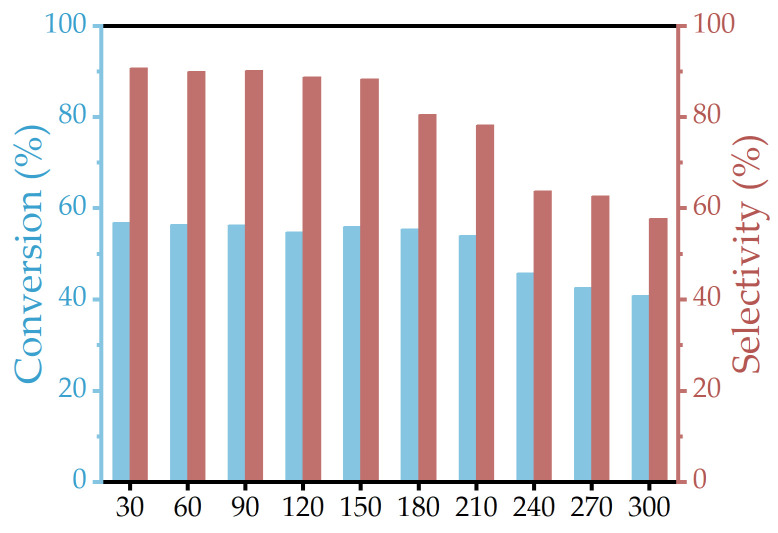
Catalytic stability of the 2%Ni/ZSM-5-138@SiO_2_-1 catalyst with time.

Figure 13 presents the time-on-stream performance of the 2% Ni/ZSM-5-138@SiO_2_-1 catalyst for *n*-heptane hydroisomerization under the optimized reaction conditions. During the initial 30–180 min of continuous operation, both the *n*-heptane conversion and isomer selectivity remained nearly constant, indicating that the catalyst possessed satisfactory catalytic stability over the initial reaction period. This result suggests that the well-balanced bifunctional catalytic sites and the core–shell architecture effectively maintained the synergy between the metallic and acidic functions during hydroisomerization. The stable catalytic performance is attributed to the maintained synergy between reduced Ni^0^ sites and zeolitic acid sites. Although Ni species are introduced as NiO precursors during catalyst preparation, the H_2_ activation process before reaction generates metallic Ni^0^ species, which participate in the continuous hydrogenation–dehydrogenation cycle during hydroisomerization.

After 180 min of reaction, the *n*-heptane conversion gradually decreased, whereas the isomer selectivity remained essentially unchanged. This observation indicates that catalyst deactivation mainly reduced the number of accessible active sites without significantly altering the intrinsic reaction pathway or product distribution. The gradual loss of catalytic activity can reasonably be attributed to coke deposition during the hydroisomerization process, which has been widely recognized as the primary deactivation mechanism for zeolite-based bifunctional catalysts [33]. The deposited coke may progressively cover both the Ni active sites and the Brønsted acid sites while partially blocking the pore channels, thereby suppressing reactant diffusion, limiting access to the active centers, and hindering the desorption of isomerized products.

Furthermore, although the SiO_2_ shell effectively passivated the external acid sites and improved the hydroisomerization performance, the presence of localized amorphous SiO_2_ domains inevitably reduced the accessibility of a fraction of the micropores. During prolonged operation, the combined effects of partial pore obstruction by the SiO_2_ shell and progressive coke accumulation further increased the diffusion resistance within the catalyst, ultimately leading to the gradual decline in catalytic activity.

To further evaluate the competitiveness of the developed Ni/ZSM-5@SiO_2_ catalyst, its catalytic performance was compared with catalysts reported for *n*-heptane hydroisomerization (Table 3). Although direct comparison is complicated by differences in metal species, reaction conditions, and catalyst structures, the comparison provides useful insight into the catalytic behavior of the present system. As shown in Table 3, the Ni/ZSM-5@SiO_2_ catalyst exhibits a higher *n*-heptane conversion (91.1%) than most reported catalysts under comparable hydroisomerization conditions. Although the isomer selectivity is slightly lower than that of highly selective 0.5Pt/SAPO-11, the present catalyst achieves a favorable balance between conversion and selectivity with a significantly shorter reaction time. This performance can be attributed to the synergistic effect of Ni hydrogenation functionality, moderated acidity of ZSM-5 after silica coating, and improved accessibility of active sites. The silica layer suppresses excessive cracking by reducing the contribution of strong external acid sites, while preserving sufficient internal acidity for skeletal rearrangement.

## 3. Materials and Methods

### 3.1. Materials

All chemical reagents used in this study were purchased from Tianjin Chemical Reagent Co., Ltd. (Tianjin, China), including aluminum isopropoxide (C_9_H_21_AlO_3_), tetraethyl orthosilicate (TEOS), tetrapropylammonium hydroxide (TPAOH), cetyltrimethylammonium bromide (CTAB), aqueous ammonia (25%), ethanol, *n*-heptane (C_7_H_16_), and nickel(II) nitrate hexahydrate (Ni(NO_3_)_2_·6H_2_O). Deionized water was prepared in-house.

### 3.2. Methods

Synthesis of ZSM-5 Zeolite: ZSM-5 zeolites with different Si/Al molar ratios (SAR = 98, 118, 138, and 158) were synthesized via a conventional hydrothermal method. The precursor gel was prepared with the molar composition of 1 C_9_H_21_AlO_3_: x TEOS: 27 TPAOH: 77 H_2_O, where x was adjusted to obtain the desired Si/Al ratio. C_9_H_21_AlO_3_ was first dissolved in a diluted TPAOH solution under vigorous stirring. Subsequently, TEOS was slowly added dropwise, and the resulting mixture was stirred for 2 h to obtain a homogeneous precursor gel. The gel was then transferred into a Teflon-lined stainless-steel autoclave and aged at room temperature for 24 h prior to crystallization at 175 °C for 2 h. After crystallization, the product was recovered by centrifugation, thoroughly washed with deionized water and ethanol at least four times, dried overnight, and finally calcined in air at 550 °C for 5 h to remove the organic template.

Synthesis of ZSM-5-x@SiO_2_-y: Core–shell ZSM-5-x@SiO_2_-y composites were synthesized through a surfactant-assisted silica coating process. Typically, 2.0 g of ZSM-5 zeolite was ultrasonically dispersed for 1 h, followed by the addition of 1.44 g CTAB, 120 mL ethanol, 160 mL deionized water, and 2 mL aqueous ammonia. Subsequently, 2.0 g of TEOS was introduced into the suspension, which was maintained at 45 °C for 600 min to allow the growth of the silica shell. The resulting solid was collected by centrifugation, washed repeatedly with deionized water and ethanol, dried, and calcined in air at 550 °C for 5 h with a heating rate of 2 °C/min. The obtained samples were denoted as ZSM-5-x@SiO_2_-y, where x represents the Si/Al ratio of the ZSM-5 core (x = 98, 118, 138, and 158), and y denotes the TEOS/ZSM-5 mass ratio (y = 0.5, 1, 2, and 3).

Preparation of Ni/ZSM-5-x@SiO_2_-y Catalysts: Ni-loaded core–shell catalysts were prepared using a conventional incipient wetness impregnation method. Appropriate amounts of Ni(NO_3_)_2_·6H_2_O were dissolved in deionized water to obtain Ni loadings of 1, 2, and 3 wt%, respectively. The resulting solution was slowly impregnated onto the corresponding ZSM-5-x@SiO_2_-y support and stirred at room temperature for 48 h. The impregnated samples were subsequently dried under ambient conditions and calcined in air at 550 °C for 4 h, yielding the final γ% Ni/ZSM-5-x@SiO_2_-y catalysts. Since this study primarily focuses on catalyst structure regulation and catalytic performance optimization rather than zeolite synthesis efficiency evaluation, the product yield was not systematically investigated.

### 3.3. Catalyst Characterization

The crystalline structures of the catalysts were characterized by XRD using a Rigaku D/MAX-2200 diffractometer (Rigaku Corporation, Tokyo, Japan) with Cu Kα radiation. FT-IR spectra were recorded on a Thermo Nicolet AVATAR-360 spectrometer (Thermo Fisher, Waltham, MA, USA). The textural properties, including the specific surface area, pore volume, and pore size distribution, were determined from N_2_ adsorption–desorption isotherms measured at −196 °C on a Micromeritics ASAP 2020 analyzer (Micromeritics, Norcross, GA, USA). Before measurement, all samples were degassed under vacuum at an appropriate temperature. The specific surface area and pore size distribution of the composite zeolites were calculated using the BET equation and BJH model, respectively. The microporous surface area and micropore volume were further determined by the t-plot method. SEM was carried out on a Zeiss SIGMA (Zeiss, Jena, Germany), while the microstructure and elemental distribution were investigated by TEM and EDS on a JEOL JEM-2100PLUS microscope (JEOL, Tokyo, Japan). The surface acidity was evaluated by NH_3_-TPD using a Micromeritics AutoChem II 2920 chemisorption analyzer (Micromeritics, Norcross, GA, USA). The surface elemental composition and chemical states were analyzed by XPS on a Thermo Scientific ESCALAB 250Xi spectrometer (Thermo Fisher, Waltham, MA, USA).

### 3.4. Catalytic Evaluation

The hydroisomerization of *n*-heptane was carried out in a laboratory-built fixed-bed continuous-flow reactor under atmospheric pressure to evaluate the catalytic performance of the prepared catalysts. Prior to the catalytic tests, the catalyst was pelletized, crushed, and sieved to obtain particles with a size of 60–80 mesh. Approximately 0.20 g of catalyst was loaded into the center of a quartz tubular reactor between layers of quartz sand, with quartz wool used to support the catalyst bed. The catalyst bed height was approximately 2 cm. Before the reaction, the catalyst was reduced under a hydrogen flow at the desired reduction temperature for a specified period. The reactor was subsequently cooled to the reaction temperature at a rate of 2 °C/min, after which *n*-heptane was introduced into the reactor. The hydroisomerization reaction was performed at a weight hourly space velocity of 6.8 h^−1^ with an H_2_/*n*-heptane molar ratio of approximately 2.82. Prior to reaction, the catalyst was activated by H_2_ reduction at 400 °C for 4 h under a continuous H_2_ flow of 30 mL min^−1^. The reaction products were analyzed online using a GC-7980A gas chromatograph equipped with an appropriate detector. Gas samples of approximately 3 mL were collected periodically to determine the catalytic activity and product distribution.

## 4. Conclusions

A series of core–shell Ni/ZSM-5@SiO_2_ bifunctional catalysts, consisting of a ZSM-5 core and an amorphous SiO_2_ shell, were successfully constructed and systematically investigated for the hydroisomerization of *n*-heptane. The effects of the SiO_2_ shell thickness, the Si/Al ratio of the ZSM-5 core, and the Ni loading on the catalyst structure, acidity, and catalytic performance were comprehensively evaluated. The results demonstrated that the amorphous SiO_2_ shell effectively passivated the external acid sites of ZSM-5 without compromising its intrinsic MFI framework, while simultaneously introducing a hierarchical pore structure that facilitated molecular diffusion. As a consequence, the metal–acid synergy was significantly improved, suppressing undesirable cracking reactions and promoting the selective formation of isomerized products. Among all the catalysts investigated, 2% Ni/ZSM-5-138@SiO_2_-1 exhibited the optimum catalytic performance, achieving an *n*-heptane conversion of 91.1% with an isomer selectivity of 56.7%, together with satisfactory catalytic stability during continuous operation. The improved hydroisomerization performance of Ni/ZSM-5@SiO_2_ catalysts originates from the optimized metal–acid synergy rather than merely increased acidity. The Ni species provide hydrogenation–dehydrogenation functionality, while the regulated acidic environment and hierarchical diffusion structure favor selective skeletal rearrangement and suppress excessive cracking. The findings offer valuable insights into the design of efficient catalysts for the hydroisomerization of long-chain alkanes and the production of clean gasoline.

## Figures and Tables

**Table 1:** Textural properties of all samples.

Catalyst	TEOS/ZSM-5 (g/g)	Surface Area (m^2^/g)			Pore Volume (cm^3^/g)		
		S_BET_	Smicro	Sext	Vtotal	Vmicro	Vext
ZSM-5-138	∞	395.33	246.30	149.02	0.18	0.11	0.07
ZSM-5-138@SiO_2_	0.5	422.41	143.96	278.65	0.31	0.10	0.21
ZSM-5-138@SiO_2_	1	500.16	106.09	394.07	0.36	0.08	0.28
ZSM-5-138@SiO_2_	2	498.99	45.87	453.12	0.36	0.07	0.27
ZSM-5-138@SiO_2_	3	479.81	37.53	442.29	0.32	0.07	0.25
1% Ni/ZSM-5-138@SiO_2_	0.5	349.58	272.11	77.46	0.30	0.13	0.17
1% Ni/ZSM-5-138@SiO_2_	1	401.74	173.24	228.50	0.32	0.09	0.23
1% Ni/ZSM-5-138@SiO_2_	2	418.43	80.52	338.43	0.31	0.06	0.25
1% Ni/ZSM-5-138@SiO_2_	3	380.00	40.62	339.38	0.30	0.07	0.23

**Table 2:** Elemental compositions (at%) of different samples.

Sample	C (at%)	Si (at%)	Al (at%)	O (at%)	Ni (at%)
ZSM-5-138	8.81	25.97	3.07	62.15	-
ZSM-5-138@SiO_2_-1	6.61	28.27	1.16	63.96	-
2%Ni/ZSM-5-138@SiO_2_-1	8.46	26.04	1.51	63.04	0.95

**Table 3:** Comparison of catalytic performance of representative catalysts for *n*-heptane hydroisomerization.

Catalyst	Reaction Temperature (°C)	Reaction Time (h)	Conversion (%)	Isomer Selectivity (%)	Reference
Pt/ZSM-50–1	270	1.0	71.4	29.1	[34]
Pt/FeAl EUO-0.25	320	1.0	66.1	14.4	[35]
Pt/ZSM-22	360	1.1	47.7	4.8	[36]
Pt/ZSM-48	340	1.6	72.0	8.8	[37]
0.5Pt/SAPO-11	312	6.8	77.0	65.0	[38]
2% Ni/ZSM-5-138@SiO_2_-1	300	0.5	91.1	56.7	This work

## Data Availability

Data will be made available on request.
